# Association of the Triglyceride–Glucose Index with Major Adverse Cardiovascular Events in Patients with Acute Coronary Syndromes: A Systematic Review and Meta-Analysis

**DOI:** 10.3390/medicina62020360

**Published:** 2026-02-11

**Authors:** Eleni Bampali, Sotirios Chiotis, Aikaterini Zgouridou, Leonidas Koliastasis, Dimitrios Vrachatis, Dimitrios-Orestis Pavlou, Vaios Schismenos, Nikolaos Chaitidis, Antonios Antoniadis, Efstathios Pagourelias, Ioannis Doundoulakis, Vassileios P. Vassilikos, Georgios Giannopoulos

**Affiliations:** 1 Third Department of Cardiology, “Hippokration” General Hospital, Aristotle University of Thessaloniki, 54642 Thessaloniki, Greece; elinabampali@gmail.com (E.B.); sotirischg@gmail.com (S.C.); zgou.katerina@gmail.com (A.Z.); dopavlou@gmail.com (D.-O.P.); vaiosschism@gmail.com (V.S.); statpag@yahoo.gr (E.P.); vvas63@otenet.gr (V.P.V.); ggiann@auth.gr (G.G.); 2 First University Department of Cardiology, “Hippokration” General Hospital, National and Kapodistrian University of Athens, 11527 Athens, Greece; lkoliastasis@gmail.com; 3 Department of Interventional Cardiology and Electrophysiology, Evgenidio Hospital, 17563 Athens, Greece; dvrachatis@gmail.com; 4 First Department of Dermatology and Venereology, “Hippokration” General Hospital, Aristotle University of Thessaloniki, 54642 Thessaloniki, Greece; nchaitidauth@gmail.com; 5 Second Department of Cardiology, “Hippokration” General Hospital, Aristotle University of Thessaloniki, 54642 Thessaloniki, Greece; aantoniadis@gmail.com; 6 Department of Health Sciences, School of Life and Health Sciences, University of Nicosia, UNIC Athens, 16777 Athens, Greece

**Keywords:** triglyceride–glucose index, acute coronary syndrome, major adverse cardiovascular events, systematic review, meta-analysis

## Abstract

*Background and Objectives*: The triglyceride–glucose (TyG) index is an accessible surrogate marker of insulin resistance and has been increasingly investigated for its prognostic relevance in cardiovascular disease. However, evidence regarding its predictive value for major adverse cardiovascular events (MACEs) in patients with acute coronary syndromes (ACSs) remains inconsistent. This study systematically assessed the association between TyG index and the risk of MACEs in individuals with ACS. *Materials and Methods*: Following PRISMA 2020 guidelines, PubMed, ScienceDirect, and ClinicalTrials.gov were searched through October 2025. Ten observational cohort studies including 32,751 ACS patients were analyzed. Studies reporting fully adjusted hazard ratios (HRs) for the association between TyG index and MACEs were eligible. A random-effects model was used to pool effect sizes expressed as adjusted HRs per 1-unit increase in the TyG index. Heterogeneity, sensitivity analyses, publication bias assessment, and meta-regression were conducted. *Results*: Higher TyG index values were significantly associated with increased MACE risk (pooled adjusted HR 1.45, 95% CI 1.25–1.68, I^2^ = 80%). Leave-one-out analysis confirmed robustness. Meta-regression analysis suggested a stronger association in cohorts consisting exclusively of patients with type 2 diabetes mellitus, with a trend toward larger effect estimates in smaller studies, potentially contributing to the observed heterogeneity. Despite small-study effects, trim-and-fill-adjusted estimates remained significant (HR 1.26, 95% CI 1.05–1.52). *Conclusions*: An elevated TyG index is independently associated with higher MACE risk in ACS patients and may be considered as an additive metabolic risk marker in combination with established risk stratification tools, pending further prospective validation.

## 1. Introduction

Acute coronary syndromes (ACSs) represent a critical and life-threatening manifestation of coronary artery disease, encompassing a clinical spectrum that includes unstable angina (UA), non-ST-segment elevation ACS (nSTE-ACS), and ST-segment elevation myocardial infarction (STEMI) [1,2]. The underlying pathophysiological mechanism of ACS is predominantly attributed to the rupture or erosion of an unstable atherosclerotic plaque within a coronary artery, subsequently triggering thrombus formation and resulting in partial or complete occlusion of the coronary vessel [3]. Despite advances in evidence-based pharmacological and interventional therapies, patients with ACS continue to exhibit substantial residual cardiovascular risk, underscoring the need for precise risk stratification and the identification of novel, cost-effective biomarkers to improve individualized management [1,2,3,4].

Major adverse cardiovascular events (MACEs), a widely used composite endpoint that includes cardiovascular death, myocardial infarction, stroke, heart failure, and urgent revascularization [5], provide a robust measure of disease progression and prognosis in ACS. Early detection of MACEs during hospitalization may enable prompt initiation of intensive personalized treatment strategies [5].

In recent years, the triglyceride–glucose (TyG) index has gained attention as a promising biomarker of metabolic status [6]. It serves as a simple and reliable surrogate marker of insulin resistance (IR), a well-established hallmark of systemic inflammation and a key pathophysiological mechanism linking atherosclerosis, endothelial dysfunction, and thrombosis [6]. The TyG index is calculated from fasting triglyceride and fasting glucose using the following formula: TyG index = ln (fasting triglycerides (mg/dL) × fasting glucose (mg/dL)/2) [6]. Accumulating evidence indicates that elevated TyG index values are associated with an increased risk of adverse cardiovascular outcomes, particularly among individuals with established coronary artery disease or metabolic comorbidities [7,8]. However, existing studies investigating the relationship between the TyG index and MACEs in patients with ACS have reported inconsistent results, likely due to variations in study design, population characteristics, and endpoint definitions. Consequently, the association between the TyG index and MACEs in ACS remains inconclusive [6,7,8].

Considering the conflicting evidence on the prognostic value of the TyG index in ACS, this systematic review and meta-analysis seek to provide a definitive assessment of its association with MACEs. The findings are expected to clarify the clinical utility of the TyG index as a potential biomarker for cardiovascular risk stratification and therapeutic decision-making in ACS.

## 2. Materials and Methods

The study was designed according to the recommendations of the Preferred Reporting Items for Systematic Reviews and Meta-Analyses (PRISMA) statement [9] (Supplementary Table S1, Figure S1). The analysis was conducted according to a protocol registered in the PROSPERO database (ID: CRD420251166764).

### 2.1. Data Sources and Searches

A comprehensive literature search was conducted in PubMed, ScienceDirect, and ClinicalTrials.gov from database inception to October 2025. The search strategy combined controlled vocabulary and free-text keywords related to acute coronary syndromes and the triglyceride–glucose index. Bibliographies of eligible articles and relevant reviews were also manually screened to ensure completeness. A baseline search strategy was developed for PubMed and adapted for the other databases. No restrictions were placed on language or publication date. Two independent investigators (EB and AZ) performed the search to minimize discrepancies. The complete search strategy is presented in detail in Supplementary Table S2.

### 2.2. Eligibility Criteria

Studies were eligible for inclusion if they enrolled adult patients with a confirmed diagnosis of acute coronary syndrome (including STEMI, nSTE-ACS and UA) as defined by current diagnostic criteria [3], evaluated the TyG index at baseline, reported the incidence of MACEs during follow-up, and provided fully adjusted hazard ratios (HRs) with corresponding 95% confidence intervals (CIs). MACEs were defined as a composite of cardiovascular death, recurrent myocardial infarction, ischemic stroke, heart failure hospitalization, or coronary revascularization procedures. Studies were required to have a follow-up period exceeding 6 months. Studies without multivariable adjustment, lacking extractable effect size, or reporting insufficient follow-up data were excluded. In cases of overlapping cohorts, the dataset with the larger sample size was retained.

### 2.3. Study Selection and Quality Assessment

Search results were imported into EndNote X9 for reference management, and duplicate records were removed. Titles and abstracts were independently screened by two reviewers (EB and AZ), with disagreements resolved through consultation with a third reviewer (SC). Full-text articles were then assessed for methodological quality using the Newcastle–Ottawa Scale (NOS) for cohort studies, evaluating selection, comparability, and outcome domains; studies scoring ≥ 7 points were considered high-quality [10].

### 2.4. Data Extraction

Data extraction was conducted by one reviewer (EB) and independently verified by a second reviewer (SC) to ensure accuracy. Extracted data included study characteristics (first author, year of publication, country, study design, sample size, and duration of follow-up), demographic and cardiovascular risk factors of the population, univariate and multivariate effect estimates along with the 95% CIs for the outcome of interest, and the covariates included in the multivariate models.

### 2.5. Statistical Analysis

Random-effects meta-analysis of HRs was performed using restricted maximum likelihood (REML) estimation of between-study variance, with Hartung–Knapp adjustment applied to all confidence intervals to improve precision in the presence of heterogeneity. The TyG index was analyzed as a continuous variable, and pooled effect estimates were expressed as the HR for each 1-unit increase in TyG. For studies reporting HRs per 1-SD increase, effect estimates were converted to HRs per 1-unit increase to ensure comparability across studies. Assuming a log-linear association between the TyG index and outcome risk, the natural logarithm of the reported HR was divided by the corresponding SD of the TyG index reported in each study, and the resulting coefficient was exponentiated. The same transformation was applied to the lower and upper bounds of the 95% confidence intervals [11,12]. Heterogeneity was evaluated using Cochran’s Q statistic, τ^2^, and the I^2^ statistic. A 95% prediction interval was calculated to quantify the expected range of effect sizes in future clinical settings. Several complementary analyses were undertaken to evaluate robustness, including sensitivity, leave-one-out analysis, trim-and-fill correction, and visual and statistical assessment of small-study effects. Possible effect modifiers were explored through meta-regression. All analyses were conducted in R (version 4.1.1) using the meta and metafor packages [13].

## 3. Results

### 3.1. Search Results

**Figure 1:**
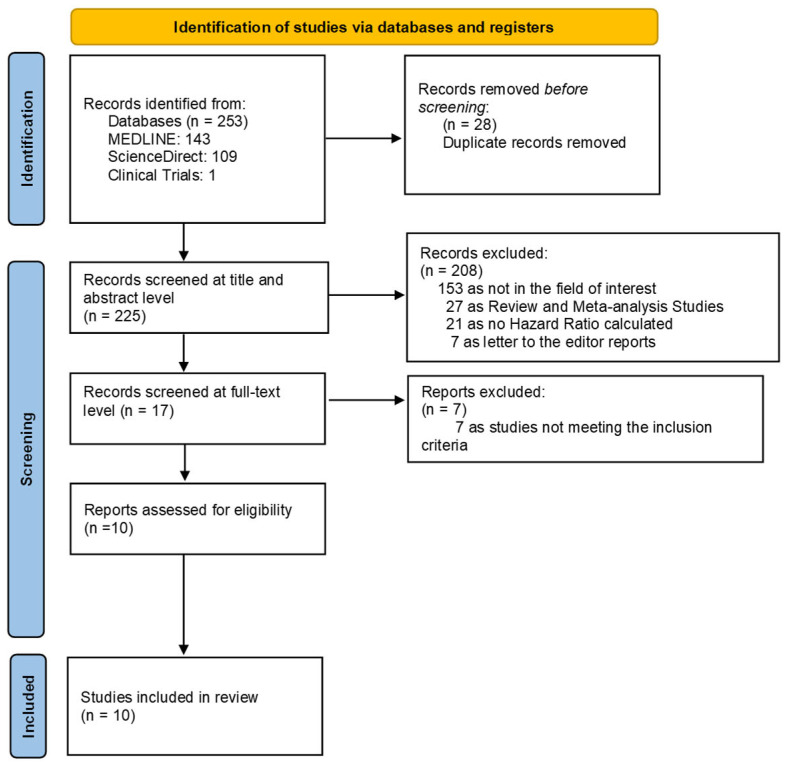
PRISMA flowchart of the study selection process.

The initial search across PubMed, ScienceDirect, and ClinicalTrials.gov yielded a total of 253 records. After removal of 28 duplicate entries, 225 unique studies underwent title and abstract screening. Of these, 208 were excluded because they were unrelated to the topic of interest, and 55 were review articles, were conference abstracts, or lacked extractable hazard ratio data. Seventeen articles were retrieved for full-text evaluation, and seven of these were excluded due to overlapping populations or not meeting the eligibility criteria. Ultimately, ten studies were included in the systematic review and quantitative meta-analysis. The study selection process is illustrated in the PRISMA flow diagram (Figure 1).

### 3.2. Study and Patient Characteristics

The ten included studies were published between 2019 and 2024 and collectively enrolled 32,751 patients with ACS [14,15,16,17,18,19,20,21,22,23]. All studies were single-center observational cohort designs, the majority conducted in China, with one large registry-based cohort from Iran and one from Israel. Follow-up duration ranged from 12 to 63 months, with a median of 24 months. Across studies, the mean age of participants was approximately 60–67 years (mean age 63.9 ± 10.8 years), with one study including only elderly adults >80 years of age. The pooled female percentage was 29%, ranging from 20% to 36%. The prevalence of type 2 diabetes mellitus (T2DM) varied substantially, with three studies including exclusively patients with T2DM. A detailed overview of study characteristics is presented in Table 1.

Despite differences in clinical setting, analytical approach, and endpoint definitions, all studies evaluated the TyG index as a prognostic biomarker for adverse cardiovascular outcomes. Several studies stratified participants according to TyG tertiles or quartiles, while all studies presented effect estimates of the TyG index as a continuous variable. Three studies originally reported hazard ratios per 1 standard deviation increase in TyG; for these, estimates were converted to per-unit increments to harmonize effect sizes across the dataset. The majority of the included studies reported both univariable and multivariable models, with only one study reporting only multivariable models. Definitions of MACEs differed modestly across studies but generally included a combination of cardiovascular death, myocardial infarction recurrence, HF hospitalization, stroke, or revascularization. A detailed summary of primary endpoints and definitions of MACEs in each study is provided in Supplementary Table S3. The covariates incorporated into multivariable adjustment were generally consistent across studies and reflected established cardiovascular risk factors. These commonly included age, sex, body mass index, hypertension, diabetes mellitus, current or prior smoking, dyslipidemia, renal dysfunction, heart failure, and prior percutaneous coronary intervention (PCI) or myocardial infarction (MI). In studies enrolling patients with acute myocardial infarction, STEMI presentation at admission was also frequently included as an adjustment variable. Additional factors occasionally considered were left ventricular ejection fraction, Killip class, reperfusion strategy, and use of evidence-based cardioprotective medications. Detailed effect estimates and the specific covariates used in each study’s adjustment model are summarized in Table 2.

### 3.3. Association of TyG Index with MACEs During Follow-Up

**Figure 2:**
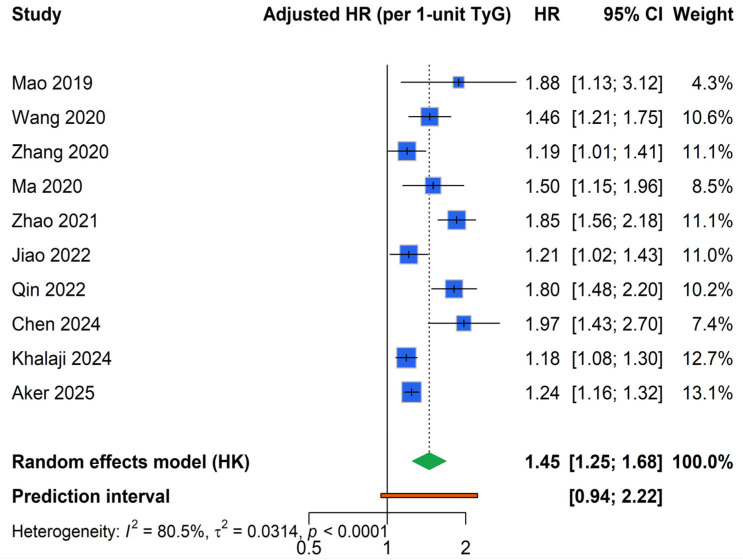
Forest plot of the pooled hazard ratio for the association of TyG index with MACEs in patients with ACS [14,15,16,17,18,19,20,21,22,23].

Across the ten included studies, higher TyG index values were associated with a significantly increased risk of major adverse cardiovascular events. Using a random-effects model, the pooled adjusted hazard ratio per 1-unit increase in TyG index was 1.45 (95% CI 1.25–1.68). Between-study heterogeneity was substantial (I^2^ = 80%, τ^2^ ≈ 0.03; *p* < 0.001). Results are presented visually in a forest plot (Figure 2).

### 3.4. Sensitivity Analysis and Meta-Regression

**Figure 3:**
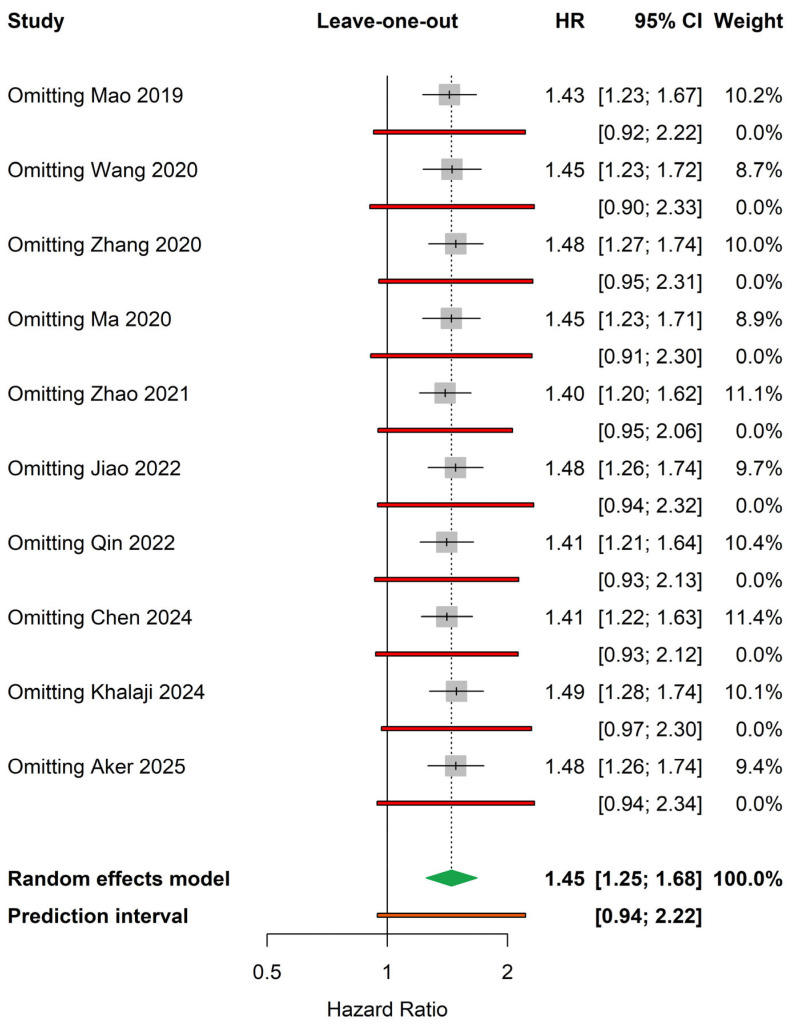
Leave-one-out sensitivity analysis [14,15,16,17,18,19,20,21,22,23].

Leave-one-out sensitivity analysis demonstrated that the removal of any individual study did not materially alter the pooled estimate or its statistical significance (Figure 3). In sensitivity analyses addressing heterogeneity in endpoint definitions, restriction to studies defining MACEs exclusively by hard clinical endpoints (death, myocardial infarction, or stroke; k = 3) yielded a pooled HR of 1.48 (95% CI 0.89–2.45, I^2^ = 90%) (Supplementary Figure S1). In a second sensitivity analysis excluding studies with the most heterogeneous composite endpoints—specifically those including cardiac hospitalization, malignant arrhythmias, or angina-driven events (included studies: k = 8), the pooled HR was 1.44 (95% CI 1.22–1.71, I^2^ = 80%) (Supplementary Figure S2). Meta-regression analysis did not identify age, follow-up duration, gender distribution, diabetes prevalence, or publication year as significant modifiers of the TyG–MACE relationship. Sample size demonstrated a trend toward moderation, with smaller studies reporting stronger associations, although this did not reach statistical significance. Notably, the association between TyG index and MACEs was significantly stronger in cohorts including exclusively patients with T2DM (β coefficient: 0.3, *p* = 0.006) (Supplementary Table S3).

### 3.5. Publication Bias and Small-Study Effects

Visual inspection of the funnel plot demonstrated noticeable asymmetry (Supplementary Figure S3). This was supported by Egger’s regression test, which indicated statistically significant small-study effects (t = 2.57, df = 8; *p* = 0.03). To account for potential publication bias, the trim-and-fill method was applied, which imputed additional studies to the left side of the funnel plot. The resulting adjusted pooled estimate remained statistically significant (HR = 1.26, 95% CI 1.05–1.52), confirming that the association between higher TyG index and increased risk of MACEs persisted even after conservative correction for small-study effects (Supplementary Figure S4).

### 3.6. Risk of Bias and Grading of Evidence

The methodological quality of the included cohort studies was evaluated using the Newcastle–Ottawa Scale (NOS). Overall, the studies demonstrated a low risk of bias, with total NOS scores ranging from 8 to 9 out of a maximum of 9 points. All studies achieved full scores in cohort representativeness, selection of non-exposed groups, ascertainment of exposure, and confirmation that outcomes were not present at baseline. Most studies also demonstrated adequate adjustment for confounders and sufficient duration and completeness of follow-up, indicating robust methodological rigor (Supplementary Table S5). Nonetheless, certainty of evidence assessed via the GRADE approach was rated as very low, primarily due to inconsistency and potential publication bias (Supplementary Table S6).

## 4. Discussion

In this systematic review and meta-analysis of ten cohort studies totaling 32,751 patients with ACS, we found that higher TyG index values were independently associated with an increased risk of major adverse cardiovascular events (MACEs) during follow-up. Notably, this is the first systematic review including a large pooled sample of more than 32,000 patients with ACS drawn from contemporary clinical cohorts, enhancing the precision and statistical power of the findings. In addition, we synthesized only fully adjusted hazard ratios, enabling evaluation of the independent prognostic value of the TyG index beyond traditional cardiovascular risk markers. The pooled adjusted HR per 1-unit increase in TyG was 1.45 (95% CI 1.25–1.68), indicating that TyG is a meaningful prognostic marker in the post-ACS population. This relationship remained robust in most sensitivity analyses and persisted following trim-and-fill correction, supporting the validity of the association.

The TyG index was originally proposed as a simple surrogate marker for IR and has been shown to correlate strongly with the euglycemic–hyperinsulinemic clamp, the gold-standard measure of IR [24,25]. Insulin resistance promotes endothelial dysfunction, oxidative stress, inflammatory cytokine activation, and pro-thrombotic signaling, all of which accelerate atherosclerotic plaque progression and instability [26,27]. In the setting of ACS, these pathophysiological mechanisms may be particularly detrimental by impairing microvascular function and myocardial metabolic recovery after ischemic injury, thereby increasing vulnerability to recurrent ischemic events.

Recent evidence has increasingly linked higher TyG levels to adverse cardiovascular outcomes across various clinical settings, including stable coronary artery disease, heart failure, and ischemic stroke [28,29,30]. In ACS specifically, elevated TyG has been associated with increased no-reflow risk, impaired microvascular reperfusion, and higher rates of recurrent ischemic events [31,32]. Our findings confirm and extend these observations by demonstrating that the risk attributable to TyG is consistent across diverse ACS cohorts and persists after adjustment for conventional cardiometabolic risk factors.

Notably, the association between TyG and MACEs appeared stronger in populations with established diabetes mellitus. This may reflect the amplified impact of insulin resistance-mediated endothelial dysfunction, altered myocardial substrate metabolism, and chronic low-grade inflammation in diabetic individuals [33,34,35]. Thus, TyG may serve as both a prognostic indicator and a potential metabolic risk stratification tool in ACS, particularly among patients with type 2 diabetes.

From a clinical standpoint, the TyG index is attractive due to its simplicity and reliance on routinely available fasting triglyceride and glucose measurements. Nevertheless, its prognostic relevance must be interpreted in the context of established risk markers routinely used in ACS, including cardiac troponins, natriuretic peptides such as NT-proBNP, inflammatory biomarkers like high-sensitivity C-reactive protein, and validated risk scores such as GRACE or TIMI. Importantly, none of the included studies formally evaluated whether TyG improves risk prediction beyond these established markers using discrimination or reclassification metrics (e.g., C-statistics or net reclassification indices). As a result, it remains unclear whether TyG provides incremental, clinically actionable prognostic information or primarily reflects metabolic risk already captured by existing biomarkers and risk models. Future large-scale prospective studies incorporating head-to-head comparisons and formal incremental prognostic analyses are therefore warranted to determine whether TyG represents an independent risk marker with additive clinical value or a potentially modifiable therapeutic target.

In addition, despite its apparent prognostic relevance, the clinical implementation of the TyG index is currently limited by the absence of validated cut-off values for risk stratification in patients with ACS. Across the included studies, TyG was treated as a continuous variable or categorized using study-specific tertiles, quartiles, or thresholds, resulting in limited clinical interpretability. Although higher TyG categories were consistently associated with worse outcomes, no consensus cut-off values have been established or prospectively validated. Furthermore, none of the available studies evaluated the integration of TyG into established ACS risk scores, such as GRACE or TIMI, nor did they assess whether adding TyG improves model discrimination, calibration, or reclassification. Defining clinically meaningful cut-off values and formally testing the incorporation of TyG into existing risk prediction tools therefore represent essential steps before the TyG index can be considered a ready-to-use biomarker in routine clinical practice.

This study has several limitations that should be acknowledged. First, all included studies were observational cohort designs; therefore, the present findings demonstrate an association rather than a causal relationship between the TyG index and adverse cardiovascular outcomes, and residual confounding cannot be excluded despite multivariable adjustment. Second, although most studies used broadly comparable definitions of MACEs, differences in endpoint composition and follow-up duration likely contributed to the substantial between-study heterogeneity observed. Sensitivity analyses addressing heterogeneity in endpoint definitions yielded directionally consistent results; however, heterogeneity remained high across these analyses, and restriction to studies using hard clinical endpoints resulted in loss of statistical significance, likely reflecting limited statistical power due to the small number of eligible studies and persistent between-study variability rather than absence of an association. Moreover, meta-regression analyses suggested that part of this heterogeneity may be related to differences in study size and metabolic risk profiles, with a stronger association observed in cohorts consisting exclusively of patients with type 2 diabetes mellitus and a trend toward larger effect estimates in smaller studies; however, these findings should be interpreted cautiously. Third, two studies originally reported the association between TyG and outcomes per one standard deviation increase; although these values were converted to per-unit increments using established log-scale transformation methods to improve comparability, slight scaling imprecision cannot be excluded. Fourth, the majority of included studies were conducted in Asian populations; therefore, the observed effect size may not be directly generalizable to Western cohorts, where differences in metabolic phenotypes, obesity prevalence, and cardiovascular risk profiles could influence the magnitude of the association between the TyG index and adverse outcomes. Fifth, although we identified evidence of small-study effects through Egger’s regression test, the association between TyG and MACEs remained robust after trim-and-fill correction, suggesting that publication bias alone does not explain the observed findings; nonetheless, its presence should be interpreted with caution. Finally, we may have excluded studies that reported alternative effect measures such as odds ratios or risk ratios; however, hazard ratios are the most appropriate metric for time-to-event outcomes and therefore provide the most valid basis for pooled analysis in this clinical setting.

## 5. Conclusions

In summary, this systematic review and meta-analysis demonstrate that a higher TyG index is independently associated with an increased risk of MACEs among patients with ACS. The strength and consistency of this association across diverse clinical cohorts, together with the simplicity and routine availability of the TyG index, suggest that it may be considered as an additive risk marker in combination with established prognostic scores, such as GRACE risk score, for improving post-ACS risk stratification, particularly in patients with underlying metabolic dysfunction such as diabetes. However, given the observational nature of the available evidence, further randomized controlled trials and interventional studies are needed to determine whether targeted modification of insulin resistance or reductions in the TyG index can translate into improved cardiovascular outcomes.

## Figures and Tables

**Table 1:** Baseline characteristics of the included studies.

Author/Year	Origin	Subjects		Design	N	Median Follow Up [IQR] (Months)
Mao et al., 2019 [14]	Single-center, Chongqing, China	nSTE-ACS patients undergoing PCI		NR	438	12
Ma et al., 2020 [15]	Single-center, Beijing, China	T2DM patients with ACS (nSTE-ACS, STEMI, UA) undergoing PCI		Retro	776	30 [24–26]
Zhang et al., 2020 [16]	Single-center, Beijing, China	AMI (nSTE-ACS, STEMI)		Retro	3181	33.3 [13.8–49.8]
Wang et al., 2020 [17]	Single-center, Tianjin, China	T2DM patients with ACS (nSTE-ACS, STEMI, UA)		Retro	2531	36
Zhao et al., 2021 [18]	Single-center, Beijing, China	T2DM patients with nSTE-ACS undergoing PCI		NR	2107	24
Jiao et al., 2022 [19]	Single-center, Beijing, China	ACS patients >80 years of age		NR	662	63 [51–74]
Qin et al., 2022 [20]	Single-center, Zhengzhou, China	T2DM patients with ACS undergoing PCI		Retro	899	23 [1–36] *
Khalaji et al., 2024 [21]	Single-center, Tehran, Iran	ACS patients undergoing PCI		Retro	13,542	12.6 [10.4–19.6]
Chen et al., 2024 [22]	Single-center, Sichuan, China	ACS patients undergoing PCI		Retro	986	30
Aker et al., 2025 [23]	Single-center, Haifa, Israel	Patients undergoing ICA		Pro	Total: 12,165 ACS: 7.629	73.2 [38.4–114]
Summary					32,751	24
**Author/Year**	**Mean Age ± SD (years)**	**Gender Female, n (%)**	**T2DM, n (%)**	**Inclusion Criteria**	**Exclusion Criteria**	**Primary** **Endpoints**
Mao et al., 2019 [14]	61 ± 11.9	143 (33)	143 (33)	nSTE-ACS patients with complete clinical information with estimated eGFR ≥ 60 mL/min/1.73 m^2^ at admission who underwent ICA	Non-obstructive CAD primary CM, VHD, hepatic dysfunction, significant infection, thyroid and adrenal cortex dysfunction, ADs, CA, hematologic disorders, surgery or trauma 3 months prior to participation, patients taking statins and TG-lowering medication before the onset of nSTE-ACS	MACEs
Ma et al., 2020 [15]	61 ± 10	216 (28)	776 (100)	Patients with T2DM and ACS who underwent ICA and were treated with primary or elective PCI	Prior CABG, cardiogenic shock, LVEF < 30%, renal dysfunction with creatinine clearance (CrCl) < 15 mL/min or chronic dialysis, BMI > 45 kg/m^2^, suspected FHTG, missing follow-up data	Composite endpoint of cardiovascular events
Zhang et al., 2020 [16]	63.3 ± 11.9	772 (24)	1231 (39)	Patients who diagnosed with AMI and underwent ICA	Acute infectious disease, rheumatic disease, hematological disease, neoplastic disease, severe VHD or CHD, lacking clinical or follow-up data	MACEs
Wang et al., 2020 [17]	66.3 ± 6.8	1116 (44)	2531 (100)	Insulin-using patients with T2DM or under hypoglycemic medications, or those with an FBG ≥ 7.0 mmol/L or a 2 h plasma glucose level on their OGTT ≥ 11.1 mmol/L	Severe VHD, severe CHD, acute infection, CA, severe hepatic or renal dysfunction, nutritional derangements, incomplete clinical data	MACEs
Zhao et al., 2021 [18]	60 ± 9	656 (31)	721 (34)	Patients aged ≥18 years who diagnosed with nSTE-ACS or UA and successfully underwent PCI and experienced no MACE during hospitalization	Missing baseline data, prior CABG, suspected FHTG, BMI > 45 kg/m^2^, taking TG medications before admission, T1DM, acute or chronic infection diseases, active bleeding (except for menstruation), situations requiring long-term OACs, cardiogenic shock, severe HF, MCS, severe hepatic dysfunction or active hepatic diseases, severe renal dysfunction with or renal replacement therapy, CA or life expectancy <1 year, unwilling or unable to complete the follow-up after discharge	MACCEs
Jiao et al., 2022 [19]	81.8 ± 2.1	186 (28)	231 (35)	ACS patients aged > 80 years	Severe VHD, PH, severe liver insufficiency, RA, infectious diseases, malignant tumors—(BMI) ≥ 45 kg/m^2^, FHTG, neuropsychiatric disorders, lost to follow-up	All-cause death; MACEs
Qin et al., 2022 [20]	60 ± 10	316 (35)	899 (100)	Patients with ACS and T2DM who underwent PCI	Age < 18 years old, HCM, CHD, CA, hyperthyroidism, anemia Ads, incomplete clinical records	MACEs
Khalaji et al., 2024 [21]	63 ± 11	3713 (27)	13,542 (040)	Patients with ACS	Lack of data on FPG and TG, lack of follow-up data	MACCEs and components
Chen et al., 2024 [22]	66.6 ± 11.4	279 (28)	355 (36)	ACS patients undergoing PCI	Prior CABG, severe SHD severe hepatic/respiratory/renal insufficiency, hematological and solid tumor with limited life expectancy, death during hospitalization, incomplete medical data	MACEs
Aker et al., 2025 [23]	65.3 ± 10.3	NR	NR	Patients aged 45–85 years underwent ICA	Lack of data on main parameters < 6 months pre-hospitalization, aged < 45 years, aged > 85 years, without CAD	MACEs
Summary	63.9 ± 10.8	7397/25.122 (29)	12,318/25.122 (49)			

* Median, [range]. Abbreviations: ACS, acute coronary syndromes; AD, autoimmune disease; AMI, acute myocardial infarction; BMI, body mass index; CABG, coronary artery bypass graft; CA, cancer; CAD, coronary artery disease; CHD, congenital heart disease; CM, cardiomyopathy; eGFR, estimated glomerular filtration rate; FBG, fasting blood glucose; FHTG, familial hypertriglyceridemia; HF, heart failure; ICA, invasive coronary angiography; IQR, interquartile range; LVEF, left ventricular ejection fraction; MACE, major adverse cardiovascular events; MACCE: major adverse cardiac and cerebrovascular events: MCS, mechanical circulatory support; N, number; nSTE-ACS, non-ST-elevation acute coronary syndrome; NR, not reported; OAC, oral anticoagulants; PCI, percutaneous coronary intervention; Pro, prospective; Retro, retrospective; SD, standard deviation; STEMI, ST-elevation myocardial infarction; T1DM, type 1 diabetes mellitus; T2DM, type 2 diabetes mellitus; TG, triglyceride; UA, unstable angina; VHD, valvular heart disease.

**Table 2:** Effect sizes of the included studies along with covariates from the multivariable models.

Author, Year	Effect Size of TyG Index as a Continuous Variable (95% CI)	Per 1 Unit/Per 1 SD	Covariates in Multivariate Models
Mao et al., 2019 [14]	1.87 (1.13–3.12)	Per 1 Unit	Previous PCI/CABG
Ma et al., 2020 [15]	1.5 (1.16–1.99)	Per 1 Unit	Age, BMI, DBP, HDL, glycosylated hemoglobin, sex, current smoking, daily drinking, PAD, CKD, HF, previous MI/PCI, use of insulin and/or oral antidiabetic agents at discharge, coronary artery disease severity, presence of lesions > 20 mm long, use of drug-coated balloon, and complete revascularization
Zhang et al., 2020 [16]	1.19 (1.01–1.41)	Per 1 Unit	Age, gender, DM, HT, previous MI, albumin, eGRF, TGs, LVEF, multi-vessel/LM
Wang et al., 2020 [17]	1.45 (1.12–1.75)	Per 1 Unit	Age, previous MI, LVEF, CRP, statin use
Zhao et al., 2021 [18]	1.84 (1.56–2.18)	Per 1 SD	Smoking history, HT, T2DM, previous MI/PCI/stroke, clinical diagnosis, TC, hs-CRP, eGFR, HbA1c, LVEF, ACEI/ARB
Jiao et al., 2022 [19]	1.21 (1.02–1.43	Per 1 SD	Age, gender male, BMI, SBP/DBP, LVEF, HT, DM, previous MI, stroke, CKD, smoking, TC, TG, ldl, hdl, eGFR, fbg, UA, aspirin, clopidogrel, statin, b-blocker, ACEI/ARB, lm lesion, multi-vessel lesion
Qin et al., 2022 [20]	1.8 (1.47–2.20)	Per 1 Unit	Cr, wbc, neut, fibrinogen, LVEF, DM duration, GRACE SCORE
Khalaji et al., 2024 [21]	1.18 (1.07–1.3)	Per 1 Unit	Age, sex, LVEF, HTN, BMI, waist circumference, LDL-C, HDL-C, Cr hemoglobin, family history of CAD, cigarette smoking, opium, type of ACS (STEMI, NSTEMI, or UA), and past medical histories of CHF, valvular heart disease, cerebrovascular disease, CPR, previous CABG/PCI, AF, SA
Chen et al., 2024 [22]	1.96 (1.43–2.69)	Per 1 Unit	Age, sex, BMI, HT, DM, smoking, previous PCI, serum creatinine, diuretics, Fib, AMI, LVEF
Aker et al., 2025 [23]	1.14 (1.1–1.19)	Per 1 SD	Age (TYG index only), sex, hyperlipidemia, HT, obesity, DM, smoking status, CKD, previous MI, PAD, cerebrovascular disease

Abbreviations: ACS, acute coronary syndrome; ACEI, angiotensin converting enzyme inhibitor; ARB, angiotensin II receptor blockers; AF, atrial fibrillation; AMI, acute myocardial infarction; B-Blocker, beta blocker; BMI, body mass index; CABG, coronary artery bypass graft; CAD, coronary artery disease; CHF, congestive heart failure; CKD, chronic kidney disease; Cr, creatinine; CRP, C-reactive protein; DBP, diastolic blood pressure; DM, diabetes mellitus; eGFR, estimated glomerular filtration rate; FBG, fasting blood glucose; GRACE SCORE, global registry for acute coronary syndromes score; HbA1, hemoglobin A1; HDL, high-density lipoprotein; HF, heart failure; HT, hypertension; LDL, low-density lipoprotein; LM, left main; LVEF, left ventricular ejection fraction; MI, myocardial infarction; Neut, neutrophils; NSTEMI, non-ST elevation myocardial infarction; PAD, peripheral artery disease; PCI, percutaneous coronary intervention; SBP, systolic blood pressure; SD, standard deviation; STEMI, ST-elevation myocardial infarction; TC, total cholesterol; TyG index, triglyceride–glucose index; TG, triglycerides; UA, unstable angina; WBC, white blood cell.

## Data Availability

The data underlying this article will be shared on reasonable request to the corresponding author.

## Supplementary Materials

The following supporting information can be downloaded at: https://www.mdpi.com/article/10.3390/medicina62020360/s1, Table S1: Preferred Reporting Items for Systematic reviews and Meta-Analysis (PRISMA) checklist, Table S2: Search terms, Table S3: Definitions of primary endpoints of the included studies, Table S4: Meta-regression analysis, Table S5: Quality assessment using the Newcastle-Ottawa Scale (NOS), Table S6: Grading of evidence for the primary outcome, Figure S1: Sensitivity analysis of studies with hard clinical outcomes, Figure S2: Sensitivity analysis excluding studies with heterogenous study endpoints, Figure S3: Funnel plot of asymmetry, Figure S4: Trim-and-fill method for publication bias.

## Abbreviations

The following abbreviations are used in this manuscript:

ACS	Acute coronary syndromes
ACEI/ARB	Angiotensin-converting enzyme inhibitor/angiotensin receptor blocker
AF	Atrial fibrillation
AMI	Acute myocardial infarction
BMI	Body mass index
CABG	Coronary artery bypass grafting
CAD	Coronary artery disease
CHF	Congestive heart failure
CKD	Chronic kidney disease
Cr	Creatinine
CRP	C-reactive protein
DBP	Diastolic blood pressure
DM	Diabetes mellitus
eGFR	Estimated glomerular filtration rate
FBG	Fasting blood glucose
GRACE	Global Registry of Acute Coronary Events
HbA1c	Glycated hemoglobin
HDL	High-density lipoprotein cholesterol
HF	Heart failure
HR	Hazard ratio
ICA	Invasive coronary angiography
IR	Insulin resistance
IQR	Interquartile range
LDL	Low-density lipoprotein cholesterol
LVEF	Left ventricular ejection fraction
MACCE	Major adverse cardiac and cerebrovascular events
MACEs	Major adverse cardiovascular events
MI	Myocardial infarction
OGTT	Oral glucose tolerance test
PAD	Peripheral artery disease
PCI	Percutaneous coronary intervention
PRISMA	Preferred Reporting Items for Systematic Reviews and Meta-Analyses
SD	Standard deviation
SBP	Systolic blood pressure
STEMI	ST-segment elevation myocardial infarction
T2DM	Type 2 diabetes mellitus
TC	Total cholesterol
TG	Triglycerides
TyG	Triglyceride–glucose index
UA	Unstable angina
WBC	White blood cell count
