# Supplementary material for: Association of the Triglyceride–Glucose Index with Major Adverse Cardiovascular Events in Patients with Acute Coronary Syndromes: A Systematic Review and Meta-Analysis
Source: Medicina (Kaunas). 2026 Feb 11;62(2):360. doi: 10.3390/medicina62020360 (PMC12941757; doi:10.3390/medicina62020360)
Supplement: Supplementary file 1 [file medicina-62-00360-s001.zip › medicina-4118369-supplementary.pdf]

Supplementary material:

Association of Triglyceride-Glucose Index with Major Adverse Cardiac Events Patients with Acute Coronary Syndromes: A Systematic Review and Meta-Analysis

Contents

Supplementary Table S1: Preferred Reporting Items for Systematic reviews and Meta-Analysis (PRISMA) checklist 2
Supplementary Table S2: Search terms 5
Supplementary Table S3: Definitions of primary endpoints of the included studies 7
Supplementary Table S4: Meta-regression analysis 8
Supplementary Figure S1: Sensitivity analysis of studies with hard clinical outcomes. 9
Supplementary Figure S2: Sensitivity analysis excluding studies with heterogenous study endpoints. 10
Supplementary Figure S4: Trim-and-fill method for publication bias........ 12
Supplementary Table S5: Quality assessment using the Newcastle-Ottawa Scale (NOS) 13
Supplementary Table S6: Grading of evidence for the primary outcome 14

Supplementary Table S1: Preferred Reporting Items for Systematic reviews and Meta-Analysis (PRISMA) checklist

Section/topic	#	Checklist item	Reported on page #
**TITLE**			
Title	1	Identify the report as a systematic review, meta-analysis, or both.	1
**ABSTRACT**			
Structured summary	2	Provide a structured summary including, as applicable: background; objectives; data sources; study eligibility criteria, participants, and interventions; study appraisal and synthesis methods; results; limitations; conclusions and implications of key findings; systematic review registration number.	1
**INTRODUCTION**			
Rationale	3	Describe the rationale for the review in the context of what is already known.	2
Objectives	4	Provide an explicit statement of questions being addressed with reference to participants, interventions, comparisons, outcomes, and study design (PICOS).	2
**METHODS**			
Protocol and registration	5	Indicate if a review protocol exists, if and where it can be accessed (e.g., Web address), and, if available, provide registration information including registration number.	2
Eligibility criteria	6	Specify study characteristics (e.g., PICOS, length of follow-up) and report characteristics (e.g., years considered, language, publication status) used as criteria for eligibility, giving rationale.	3
Information sources	7	Describe all information sources (e.g., databases with dates of coverage, contact with study authors to identify additional studies) in the search and date last searched.	3
Search	8	Present full electronic search strategy for at least one database, including any limits used, such that it could be repeated.	3

Study selection	9	State the process for selecting studies (i.e., screening, eligibility, included in systematic review, and, if applicable, included in the meta-analysis).	3
Data collection process	10	Describe method of data extraction from reports (e.g., piloted forms, independently, in duplicate) and any processes for obtaining and confirming data from investigators.	3
Data items	11	List and define all variables for which data were sought (e.g., PICOS, funding sources) and any assumptions and simplifications made.	3
Risk of bias in individual studies	12	Describe methods used for assessing risk of bias of individual studies (including specification of whether this was done at the study or outcome level), and how this information is to be used in any data synthesis.	3
Summary measures	13	State the principal summary measures (e.g., risk ratio, difference in means).	3
Synthesis of results	14	Describe the methods of handling data and combining results of studies, if done, including measures of consistency (e.g., I^2) for each meta-analysis.	3
Risk of bias across studies	15	Specify any assessment of risk of bias that may affect the cumulative evidence (e.g., publication bias, selective reporting within studies).	3
Additional analyses	16	Describe methods of additional analyses (e.g., sensitivity or subgroup analyses, meta-regression), if done, indicating which were pre-specified.	3
**RESULTS**			
Study selection	17	Give numbers of studies screened, assessed for eligibility, and included in the review, with reasons for exclusions at each stage, ideally with a flow diagram.	3,4, Figure 1
Study characteristics	18	For each study, present characteristics for which data were extracted (e.g., study size, PICOS, follow-up period) and provide the citations.	4,5, Table 1
Risk of bias within studies	19	Present data on risk of bias of each study and, if available, any outcome level assessment (see item 12).	Supplementary Table S4
Results of individual studies	20	For all outcomes considered (benefits or harms), present, for each study: (a) simple summary data for each intervention group (b) effect estimates and confidence intervals, ideally with a	11, Table 2

		forest plot.	
Synthesis of results	21	Present results of each meta-analysis done, including confidence intervals and measures of consistency.	11, Figure 2
Risk of bias across studies	22	Present results of any assessment of risk of bias across studies (see Item 15).	14
Additional analysis	23	Give results of additional analyses, if done (e.g., sensitivity or subgroup analyses, meta-regression [see Item 16]).	12, 13, Supplementary Table S3, Figure 3, Supplementary Figures S1, S2
**DISCUSSION**			
Summary of evidence	24	Summarize the main findings including the strength of evidence for each main outcome; consider their relevance to key groups (e.g., healthcare providers, users, and policy makers).	14,15
Limitations	25	Discuss limitations at study and outcome level (e.g., risk of bias), and at review-level (e.g., incomplete retrieval of identified research, reporting bias).	15
Conclusions	26	Provide a general interpretation of the results in the context of other evidence, and implications for future research.	15
**FUNDING**			
Funding	27	Describe sources of funding for the systematic review and other support (e.g., supply of data); role of funders for the systematic review.	15

Supplementary Table S2: Search terms

**Date 16.10.2025**		
Search entry Pubmed		
Search	Entry terms	Result
#1	"Triglyceride-glucose index" OR "tyg index" OR "tyg index cardiovascular "	3,458
#2	"Acute coronary syndrome" OR "acute coronary syndromes" OR "unstable angina" OR "Non-ST-elevation myocardial infarction" OR "NSTEMI" OR "ST-elevation myocardial infarction" OR "STEMI" OR "Acute Coronary Syndrome"[Mesh]	97,722
#3	#1 AND #2	143
Search entry ScienceDirect		
Search	Entry terms	Result
#1	"Triglyceride-glucose index" OR "tyg index"	1,067

#2	"Acute coronary syndrome" OR "acute coronary syndromes" OR "unstable angina" OR "Non-ST-elevation myocardial infarction" OR "NSTEMI" OR "ST-elevation myocardial infarction" OR "STEMI"	156,999
#4	#1 AND #2	109
Search entry Clinical Trials		
Search	Entry terms	Result
#1	"Triglyceride-glucose index" OR "tyg index"	11
#2	"Acute Coronary Syndromes" OR "Acute Coronary Syndrome" OR "ACS"	851
#4	#1 AND #2	1
Summary		
Combined search results from all search engines		253
After exclusion of duplicates, review articles, case reports, conference publications, and editorials.		10

Supplementary Table S3: Definitions of primary endpoints of the included studies

Author/year	Endpoint	Endpoint definition
Mao et al. 2019	MACEs	Cardiac death, nonfatal myocardial infarction, target vessel revascularization, congestive heart failure, and nonfatal stroke
Wang et al. 2020	MACEs	All-cause death, non-fatal myocardial infarction and non-fatal stroke
Zhang et al.2020	MACEs	Cardiac death, all-cause death, revascularization, cardiac hospitalization
Ma et al. 2020	Composite endpoint of cardiovascular events	All-cause mortality, non-fatal stroke, non-fatal myocardial infarction, or unplanned repeat revascularization
Zhao et al. 2021	MACCEs	Cardiac death, non-fatal myocardial infarction, and non-fatal ischemic stroke
Jiao et al. 2022	MACEs	Non-fatal acute myocardial infarction, coronary artery revascularization and all-cause mortality
Qin et al. 2022	MACEs	All-cause death, malignant arrhythmia, non-fatal myocardial infarction, target vessel reconstruction, angina pectoris requiring hospitalization, and acute heart failure
Chen et al. 2024	MACEs	All-cause death, non-fatal myocardial infarction and unplanned revascularization
Khalaji et al. 2024	MACEs	All-cause mortality, myocardial infarction, stroke, target vessel revascularization, target lesion revascularization, and coronary artery bypass grafting
Aker et al. 2025	MACEs	Myocardial infarction, ischemic stroke and all-cause death

Supplementary Table S4: Meta-regression analysis

Moderator	k	β	95% CI	p-value	R^2 (%)	Interpretation
Age (years)	10	-0.0127	-0.035 to 0.010	0.23	6.4	No significant effect
Sample size	10	-0.00001	-0.0001 to 0.0000	0.08	29.2	No significant effect
Follow-up (months)	10	-0.004	-0.011 to 0.003	0.21	2.6	No significant effect
Female (%)	9	1.199	-1.6753 to 4.0731	0.36	1.7	No significant effect
Diabetes (%)	9	0.121	-0.4576 to 0.6992	0.64	0.0	No significant effect
**Diabetes (binary)**	**10**	**0.302**	**0.1168 to 0.4878**	**0.006**	**100**	**Significant effect: stronger association in studies including patients with diabetes**
Publication year	9	-0.007	-0.1160 to 0.1010	0.87	0.0	No significant effect

Supplementary Figure S1: Sensitivity analysis of studies with hard clinical outcomes.

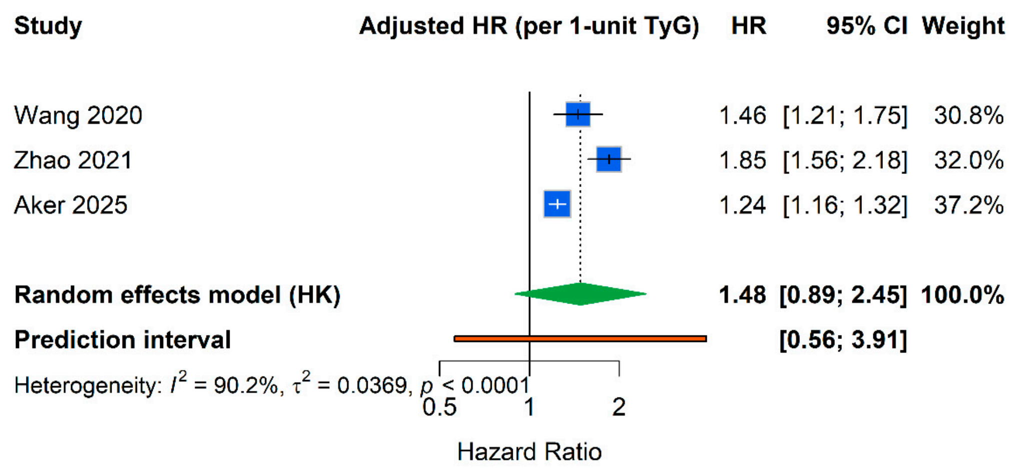

Supplementary Figure S2: Sensitivity analysis excluding studies with heterogenous study endpoints.

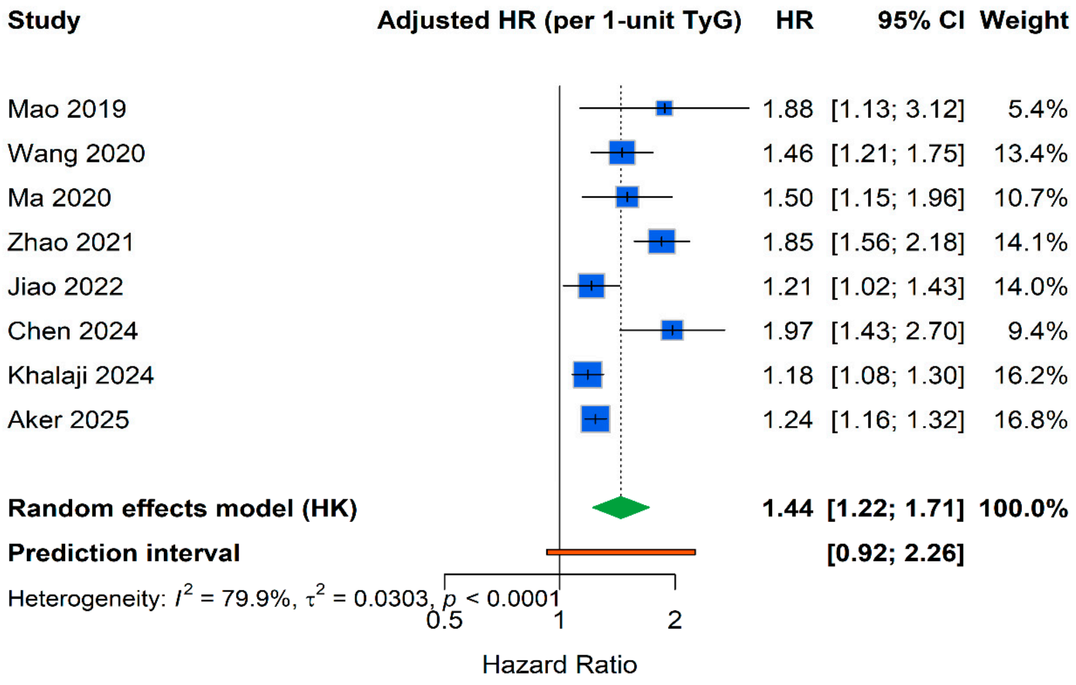

Supplementary Figure S3: Funnel plot of asymmetry.

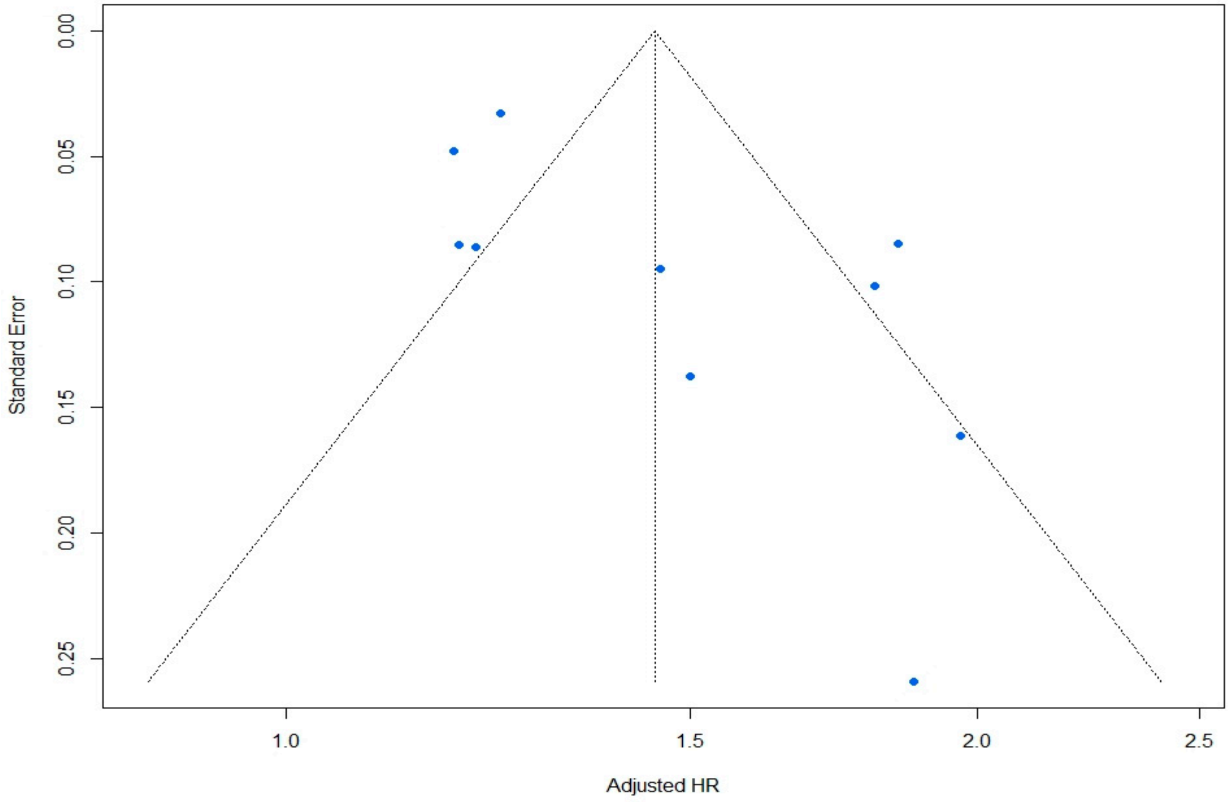

Supplementary Figure S4: Trim-and-fill method for publication bias

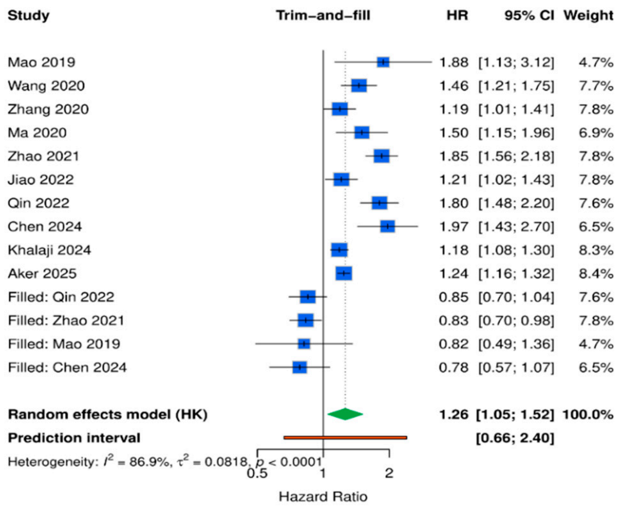

Supplementary Table S5: Quality assessment using the Newcastle-Ottawa Scale (NOS)

**Studies**	**Selection**				**Comparability**	**Exposure**			**Total Score**
	Representativeness of the Exposed Cohort	Selection of the Non-Exposed Cohort	Ascertainment of Exposure	Demonstration That Outcome of Interest Was Not Present at Start of Study		Assessment of Outcome	Was Follow-Up Long Enough for Outcomes to Occur	Adequacy of Follow Up of Cohorts	
**Mao (2019)**	*	*	*	*	**	*	*	*	9
**Wang (2020)**	*	*	*	*	**	*	*	*	9
**Zhang (2020)**	*	*	*	*	*	*	*	*	8
**Ma (2020)**	*	*	*	*	**	*	*	*	9
**Zhao (2021)**	*	*	*	*	*	*	*	*	8
**Jiao (2022)**	*	*	*	*	**	*	*	*	9
**Qin (2022)**	*	*	*	*	**	*	*	*	9
**Chen (2024)**	*	*	*	*	**	*	*	*	9
**Khalaji (2024)**	*	*	*	*	**	*	*	*	9
**Aker (2025)**	*	*	*	*	**	*	*	*	9

Supplementary Table S6: Grading of evidence for the primary outcome

Variables	No of studies	Relative effect	Certainty of evidence (GRADE)
**TyG Index**	10	HR: 1.45 (95% CI: 1.25; 1.68, I^2: 80.5%)	⊕○○○ **VERY LOW[1,2]**

CI, Confidence Interval; HR, Hazard Ratio; uHR, unadjusted Hazard Ratio

GRADE Working Group grades of evidence.
High = This research provides a very good indication of the likely effect. The likelihood that the effect will be substantially different is low.
Moderate = This research provides a good indication of the likely effect. The likelihood that the effect will be substantially different is moderate.
Low = This research provides some indication of the likely effect. However, the likelihood that it will be substantially different is high.
Very low = This research does not provide a reliable indication of the likely effect. The likelihood that the effect will be substantially different is very high.

[1] Due to publication bias
[2] Due to inconsistency
